# Comparison of carbon balances between continuous-cover and clear-cut forestry in Sweden

**DOI:** 10.1007/s13280-015-0756-3

**Published:** 2016-01-07

**Authors:** Tomas Lundmark, Johan Bergh, Annika Nordin, Nils Fahlvik, Bishnu Chandra Poudel

**Affiliations:** Department of Forest Ecology and Management, Swedish University of Agricultural Sciences, 901 86 Umeå, Sweden; Department of Forestry and Wood Technology, Linnaeus University, 351 95 Växjö, Sweden; Department of Forest Genetics and Plant Physiology, Umeå Plant Science Centre, Swedish University of Agricultural Sciences, 901 86 Umeå, Sweden; Southern Swedish Forest Research Centre, Swedish University of Agricultural Sciences, 230 53 Alnarp, Sweden

**Keywords:** Boreal, Climate change mitigation, Substitution

## Abstract

Continuous-cover forestry (CCF) has been recognized for the production of multiple ecosystem services, and is seen as an alternative to clear-cut forestry (CF). Despite the increasing interest, it is still not well described how CCF would affect the carbon balance and the resulting climate benefit from the forest in relation to CF. This study compares carbon balances of CF and CCF, applied as two alternative land-use strategies for a heterogeneous Norway spruce (*Picea abies*) stand. We use a set of models to analyze the long-term effects of different forest management and wood use strategies in Sweden on carbon dioxide emissions and carbon stock changes. The results show that biomass growth and yield is more important than the choice of silvicultural system per se. When comparing CF and CCF assuming similar growth, extraction and product use, only minor differences in long-term climate benefit were found between the two principally different silvicultural systems.

**Electronic supplementary material:**

The online version of this article (doi:10.1007/s13280-015-0756-3) contains supplementary material, which is available to authorized users.

## Introduction

An actively managed forest landscape that provides a large amount of sustained biomass yield while at the same time maintaining large standing forest carbon stocks, provides greater climate benefits in the long run compared to unmanaged forests (Nabuurs and Masera 2007; Lundmark et al. 2014). Different forest management strategies might however result in different climate benefits due to the complex interaction between forest management, carbon stored in the ecosystem and harvested wood products, and the amount of harvested products that leads to differences in substitution carbon benefit (Smyth et al. 2014).

At the stand level, the carbon balance at any given time of a managed forest is determined by the difference between the input flux of carbon (net primary production) and the output fluxes of carbon (heterotrophic respiration and leaching) together with biomass removals by harvests (Clarke et al. 2015). The long-term average annual change in carbon balance of a forest management system depends not only on the dynamics of carbon stock in the soil and standing biomass, but also on the carbon emissions related to silvicultural operations, and the level of sustained yield that can be attained. As a result of sustained forest production, some of society’s consumption will be based on renewable products reducing the net emissions of carbon to the atmosphere through the substitution of fossil-based materials. This substitution effect depends on what products that are consumed in society and substituted with forest products e.g., fossil fuels, steel, and concrete (Sathre and O’Connor 2010; Gustavsson and Sathre 2011; Poudel et al. 2011; Gustavsson et al. 2015). Several studies have shown the importance of a sustained or increased yield in actively managed forest to increase the climate benefit (Canadell and Raupach 2008; Malmsheimer et al. 2008; Poudel et al. 2012; Lundmark et al. 2014; Sievänen et al. 2014).

Intensively managed boreal forests in Finland and Sweden have high rates of productivity, low rates of natural disturbances, allowing for large transfers of forest raw material from forests to society. Forestry in these countries is largely based on clear-cut forestry (CF) with even-aged forest stands and an even age-class distribution on the landscape level (Yrjölä 2002). The carbon dynamics of CF on the stand level include a significant net biomass carbon removal from the forest stand at final harvest, rapid net carbon gain in young stands, and slower net carbon gain, after canopy closure in more mature stands (Hyvönen et al. 2007; Diochon et al. 2009). Growth of managed even-aged forest stands follows a universal pattern where current annual increment increases after stand establishment, peaks when maximum leaf area is attained, and then declines (Assmann 1970). Most often CF stands are harvested around the time when mean annual increment culminates (Möller et al. 1954). An alternative to CF is continuous-cover forestry (CCF), a silvicultural system without a clear-cut phase. CCF typically has uneven-aged stand structure and a continuously maintained forest cover, which does not follow a cyclic harvest-and-regeneration pattern on the stand level as it occurs in CF (Troup 1928; Gadow 2001). Instead, multiple selective cuttings over regular time intervals characterize CCF system. In the classical CCF silvicultural practice single-tree selection, individual trees are harvested throughout the stand to maintain an uneven-aged (and uneven-sized) stand, achieve a desired diameter distribution, and to allow establishment and ingrowth of new naturally established seedlings (Ahlström and Lundqvist 2015).

There has been a discussion for centuries on the comparison of production levels, yield, economic return, and silvicultural measures to be used in CF and CCF systems (Wallmo 1897; Holmgren 1914; Möller 1922; Troup 1928; Lundqvist 1989). The long-term trends in forest management in the Nordic countries have resulted in a completely dominant practice of the CF system (Anon. 2014a, b). Recently however, CCF has been put forward as an alternative to CF because of the provision of more ecosystem services related to a continuous forest cover (Tahvonen 2009; Kuuluvainen et al. 2012; Pukkala et al. 2012). Among those, some positive effects on the carbon dynamics has been suggested (Lindroth et al. 2012; Pukkala 2014). Despite the increasing interest, it is still not well described how CCF would affect the carbon balance and the resulting climate benefit from the forest in relation to CF.

In this paper we compare the climate benefit of CF and CCF applied as different silvicultural programs on the stand level under Nordic conditions. When doing such comparison it is important to consider time and scale perspectives (Kuuluvainen et al. 2012; Lundmark et al. 2014). A comprehensive life-cycle analysis integrating biological and technological features can help identify appropriate long-term approaches to carbon management through land use. In order to evaluate silvicultural programs in terms of climate change mitigation effectiveness, this study aims to compare the dynamics of carbon balances of CF and CCF as two alternatives for future management of an existing mature forest stand in the boreal zone in Sweden. We track carbon dynamics in standing biomass, litter on the forest floor, harvested products, and the contribution of forest biomass to substituting fossil fuels and carbon-intensive materials. Carbon balance is expressed as the combined effect of carbon sink/source effects in the forest ecosystem and harvested wood products and substitution effects.

## Materials and methods

### Clear-cut and continuous-cover forest in this study

As a starting point for this analysis, we assumed the existence of a heterogeneous Norway spruce (*Picea abies*) stand at an average fertile site in central Sweden, where the landowner could choose between doing a clear-cut and regenerate the stand artificially, or alternatively can build on the heterogeneous stand structure and develop a long-term selective cutting program to retain a continuous forest cover. The two alternatives of future forest development were simulated with growth models.

CF has even-aged stand structure that follows a cyclic harvest-and-regeneration pattern. The clear-cut silvicultural program in this study was represented by a Norway spruce forest stand that was established by planting immediately after clear-cutting of the original stand. Commercial thinning was carried out according to thinning guidelines for practical forestry in Sweden. The rotation period was set in order to optimize average forest production. Planting was carried out manually after soil scarification, commercial thinning was done by thinning harvesters, and the final cut was done using a large harvester.

CCF is based on the assumption that harvested trees are replaced by ingrowth of smaller trees, so that the total number and diameter distribution of trees is kept more or less constant over time. CCF in this study was represented by an uneven-aged Norway spruce forest managed with a single-tree selection system and was built on the same original stand as the one that was the starting point for the CF. All harvest operations were done as selective cutting by large harvesters, repeated once every decade.

### Forest growth and harvest modeling for Clear-cut and Continuous-cover forestry

Because of limited availability of empirical data, estimating biomass production of CCF under Nordic conditions is associated with uncertainty. A number of field studies where the two systems have been compared indicate a long-term production level in CCF corresponding to approximately 80 % of the mean production over a CF rotation period on comparable sites (Lundqvist 1989; Andreassen and Øyen 2002; Elfving 2006). In contrast Pukkala et al. (2009) modeled similar production levels of both systems. A majority of the existing comparisons between the two silvicultural systems are based on studies carried out in relatively mature stands. In the future, a limitation of CCF might be to rely on natural regeneration while the advancements in plant breeding and management techniques would further increase future forest production in CF (Nilsson et al. 2011).

In this study, stand development in CF was simulated with the Heureka system (Wikström et al. 2011) while stand development in CCF mainly relied on models developed by Chrimes and Lundqvist (2004). A detailed description of the simulations, site conditions, and the initial stand is found in 10.1007/s13280-015-0756-3.

Two scenarios with CF were assumed: one where only stem-wood was harvested (*CF*) and the other with a higher degree of extraction where 80 % of the residues and stumps after clear-cut was also harvested (*CF*+). The CF scenario could also be seen as the business as usual scenario. For the CCF scenarios, the simulations were set to correspond to two predefined scenarios; CCF with mean annual volume increment (MAI) corresponding to 80 % (*CCF80*) and 100 % (*CCF100*) of *CF* at equilibrium, respectively. The proportion of mortality out of total volume production in CCF was also set equal to CF. Equilibrium in CCF was met when ingrowth compensated for harvest and mortality and identical 10-year cycles were repeated over time. Only stem-wood was harvested in the CCF scenarios, thus all residues were left in the forests.

### Forest product harvest and use

It was assumed that only stem-wood was extracted in the scenarios *CF*, *CCF80*, and *CCF100*. We assumed that 95 % of the available stem-wood was extracted from the forest, and that 5 % remained in the forest as living retention trees as general concern to conservation values. In *CF*+ scenario, 95 % of available stem-wood as well as 80 % of the residues and stumps were assumed to be extracted. Also in this scenario 5 % of the available biomass remained in the forest as living retention trees.

Replacement of energy intensive products like concrete and steel as well as fossil based products results in decreased emissions of carbon dioxide (CO_2_). This kind of substitution effect influences the total carbon balance of different silvicultural systems at the landscape level as well as at the stand level. The substitution values depend on the use of forest biomass for different purposes such as construction material, bioenergy, and pulp and paper (Sathre and O’Connor 2010; Lundmark et al. 2014). For example, Sathre and O’Connor (2010) performed a meta analysis of greenhouse gas displacement factors of wood product substitution and found that most of the substitution factors in the studies were in the range of 1.0–3.0 units of fossil carbon emission avoided per unit of carbon in a wood product.

In the present study, two product utilization strategies were analyzed. In the first strategy, large diameter stem-wood was assumed to be used for production of wood construction material and small diameter stem-wood and residues were used for energy production in a combined heat and power plant only, i.e., to maximize the substitution effect. With this strategy, the estimated average substitution effect was 0.90 Mg CO_2_-eqv for each cubic meter of harvested stem-wood and biomass. In practical forestry, a significant share of the harvested biomass is used for pulp and paper, which will lower the substitution effect (Lundmark et al. 2014). In the second strategy, we used a lower estimate of the substitution effect that was 0.47 Mg CO_2_-eqv for each cubic meter of forest biomass used.

### Soil and litter carbon

A large part of the total carbon stock in a boreal forest is located belowground (Clarke et al. 2015; Piirainen et al. 2015). This carbon is found in the soil organic matter as well as in living biomass. A large part of the soil carbon is older carbon with slow turnover. All of these components should be considered when assessing the carbon balance of a forest ecosystem. Disturbances such as clear-cutting have the potential to increase decomposition and mineralization of soil carbon, thus decreasing the soil carbon stock (Jandl et al. 2007) while thinning or selective cutting might cause only small losses of carbon from the soil (Jurgensen et al. 2012). Experimental results show, however, conflicting results on the dynamics of soil organic carbon in managed forests in relation to management activities (Thiffault et al. 2011; Clarke et al. 2015) and the available information does not support firm conclusions about the long-term effects of different harvest regimes in managed boreal forests and in many cases no change have been reported (Johnson and Curtis 2001; Jandl et al. 2007; Kreutzweiser et al. 2008). We therefore assume similar dynamics of old soil carbon, as well as litter carbon originating from the period before the study period, in the different scenarios used in this study.

Litter input transfer new carbon from biomass stocks to soil carbon stocks. The litter input from living trees and mortality of trees as well as from harvest residues left in the forest during thinning and final felling were accounted for in all scenarios. We assume that the decomposition of litter produced during the study period followed the same pattern for all scenarios. Several studies in boreal forests have reported mass loss decomposition functions (Eq. 1) based on a negative exponential approach that uses a constant rate of decomposition (e.g., Hyvönen and Ågren 2001; Melin et al. 2009).

1 $$ Y_{t} = Y_{0} \cdot e^{ - kt} , $$

where *Y*_*t*_ is the mass at time *t*, *Y*_0_ the initial mass, and *k* the constant decomposition rate.

In the present study, we used specific constant rates for different biomass components to estimate the remaining fractions of all types of biomass for each year during the studied period (Table 1). The remaining fractions of all litter biomass expressed as dry matter were summed for each year to determine the total litter left in the forest and multiplied by 0.5 to convert to the carbon value. An overall summary of the scenarios in this study is presented in Table 2.

**Table 1:** The decomposition rates used in this study to determine remaining litter biomass in the forest

Biomass components	Variable	Decomposition rate (year^−1^)	Location	References
Stumps and root system	Dry biomass	0.046	Sweden	Melin et al. (2009)
Needles	Dry biomass	0.438	Sweden	Hyvönen and Ågren (2001)
Tops and branches	Dry biomass	0.070	Sweden	Hyvönen and Ågren (2001)
Stem-wood	Dry biomass	0.056	Sweden	Hyvönen and Ågren (2001)
Bark	Dry biomass	0.058	Finland	Shorohova et al. (2008)
Dead wood (snag)	Dry biomass	0.032	NW Russia	Yatskov et al. (2003)

**Table 2:** Summary of the scenarios in this study for biomass production level, silvicultural program, and harvest strategies. For abbreviations, see “Materials and methods” section

Forest management scenario	Production level (m^3^ ha^−1^)	Silvicultural program	Biomass fraction harvested
*CF*	7.01	Thinning at 45, 65 years, final harvest at 95 years	Stem-wood only
*CF*+	7.01	Thinning at 45, 65 years, final harvest at 95 years	Stem-wood, residues and stumps
*CCF100*	7.01	Selection cutting every 10 years	Stem-wood only
*CCF80*	5.61	Selection cutting every 10 years	Stem-wood only

### Total carbon balance

The total carbon balance in the present study was calculated as the sum of carbon stock changes in living tree biomass, litter, wood products stock, and carbon benefit from substitution of materials and fossil fuel. Older soil carbon stock dynamics was assumed to be the same for all scenarios and was not included in the estimate of total carbon balance since it did not affect the relationship between the scenarios.

Our system perspective was the stand level and the comparison is made over three normal rotation periods for CF. The balances for the CF scenarios were estimated for the period that started 1 year before the clear-cut of the original stand to the year before the final cut of the third rotation, a period of 285 years. The balances for the CCF scenarios were estimated for the period that started 1 year before the first selective cutting of the original stand to the year before the 29th selective cutting, a period of 290 years. To compare the long-term climate benefit, we calculate the total carbon balance of the studied system as the annual average change in carbon stocks and add that to the annual average substitution effect during the study period for each scenario.

## Results

According to the assumptions made in this study, the average annual biomass production was the same in three scenarios, i.e., *CF*, *CF*+, and *CCF100*, but was lower for the *CCF80* scenario. The biomass removal from the stand was highest for the *CF*+ scenario, where residues and stumps were also harvested (Table 3).

**Table 3:** Annual average biomass production and harvested biomass (Mg dry biomass ha^−1^ year^−1^) for the different scenarios during the study period. For abbreviations, see “Materials and methods” section

Biomass types	Forest management alternatives							
	Biomass production				Biomass removal from forest			
	*CF+*	*CF*	*CCF100*	*CCF80*	*CF+*	*CF*	*CCF100*	*CCF80*
Stem-wood and bark	2.78	2.78	2.65	2.13	2.18	2.18	2.54	2.02
Residues	1.03	1.03	1.04	0.84	0.69	0.00	0.00	0.00
Stumps	0.85	0.85	0.96	0.77	0.58	0.00	0.00	0.00
Total	4.66	4.66	4.65	3.74	3.45	2.18	2.54	2.02

For the CCF scenarios, the long-term annual average carbon stock change in living trees was close to zero while an annual net increase of 0.35 Mg C ha^−1^ year^−1^ occurred in the CF scenarios (Table 4).

**Table 4:** Annual average changes in carbon stock (Mg C ha^−1^ year^−1^) for standing biomass, litter on the forest floor, harvested products, and the annual average substitution effect (Mg C ha^−1^ year^−1^) for the different scenarios during the study period assuming a substitution effect of 0.90 Mg CO_2_-eqv for each cubic meter of harvested stem-wood. Long-term climate benefit (Mg C ha^−1^ year^−1^) is expressed as the sum of the annual average change in carbon stocks and the annual average substitution effect

Carbon stock	*CF+*	*CF*	*CCF100*	*CCF80*
Standing forest C-stock	0.35	0.35	0.01	0.00
Litter C-stock	0.01	0.02	0.05	0.04
Wood product C-stock	0.15	0.15	0.12	0.11
Substitution C-benefit	2.24	1.74	1.88	1.68
Long-term climate benefit	2.75	2.25	2.06	1.83

**Fig. 1:**
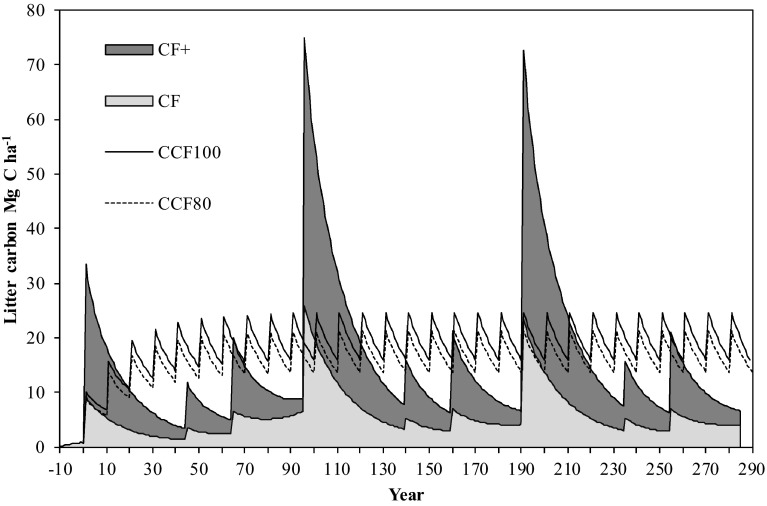
The development of litter (branches, needles, roots) carbon stock for all scenarios

The litter carbon stock in the forest varied considerably over time and between the scenarios, depending on whether residues and stumps where harvested or not (Fig. 1). The magnitude of change was higher for the CF scenarios than for the CCF scenarios (Fig. 1). The long-term average annual litter carbon stock change did not, however, vary much between scenarios, ranging 0.01, 0.02, 0.04, and 0.05 Mg C ha^−1^ year^−1^ for *CF*+, *CF*, *CCF80*, and *CCF100*, respectively (Table 4). A sensitivity analysis where the rate of decomposition in Table 1 was increased or decreased by 20 % gave only minor effects (<2 %) on the absolute values of the long-term climate benefit in Table 4 (results not shown).

**Fig. 2:**
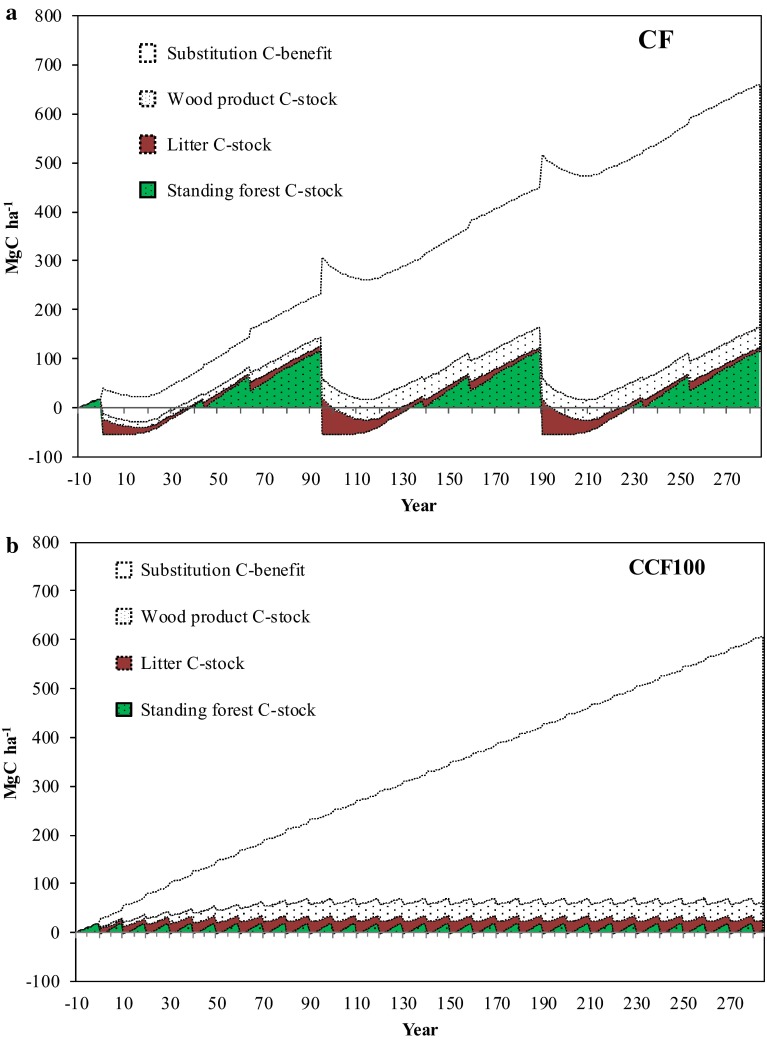
Cumulative total carbon balances in **a** *CF* and **b** *CCF100* scenarios over 285 years assuming an average substitution effect of 0.90 Mg CO_2_-eqv for each cubic meter of harvested stem-wood

There was a considerable short-term variation in carbon stock in the living biomass of trees due to annual growth rate dynamics and periodic harvest operations (Fig. 2a, b). There were small differences between scenarios in long-term carbon stock in harvested wood products.

Over the long-term, the substitution effect accounted for the largest component of the average net annual carbon balance for the different scenarios. With the assumption that forest biomass was used to maximize the substitution effect (substitution effect of 0.90 Mg CO_2_-eqv for each cubic meter of harvested biomass), at the end of the study period it accounted for 82 % of the total climate benefit for the *CF*+ scenario and 91 % for the *CCF100* scenario. Adding all components together and assuming a substitution effect of 0.90 Mg CO_2_-eqv for each cubic meter of harvested biomass, the accumulated carbon balance values for the different scenarios *CF*+, *CF*, *CCF100*, and *CCF80* during the whole study period were estimated to be 784, 642, 597, and 531 Mg C ha^−1^, respectively (Fig. 2; Table 4). With these assumptions, the average net annual carbon balance values for *CF*+, *CF*, *CCF100*, and *CCF80* were estimated to be 2.75, 2.25, 2.06, and 1.83 Mg C ha^−1^ year^−1^, respectively (Table 4).

**Fig. 3:**
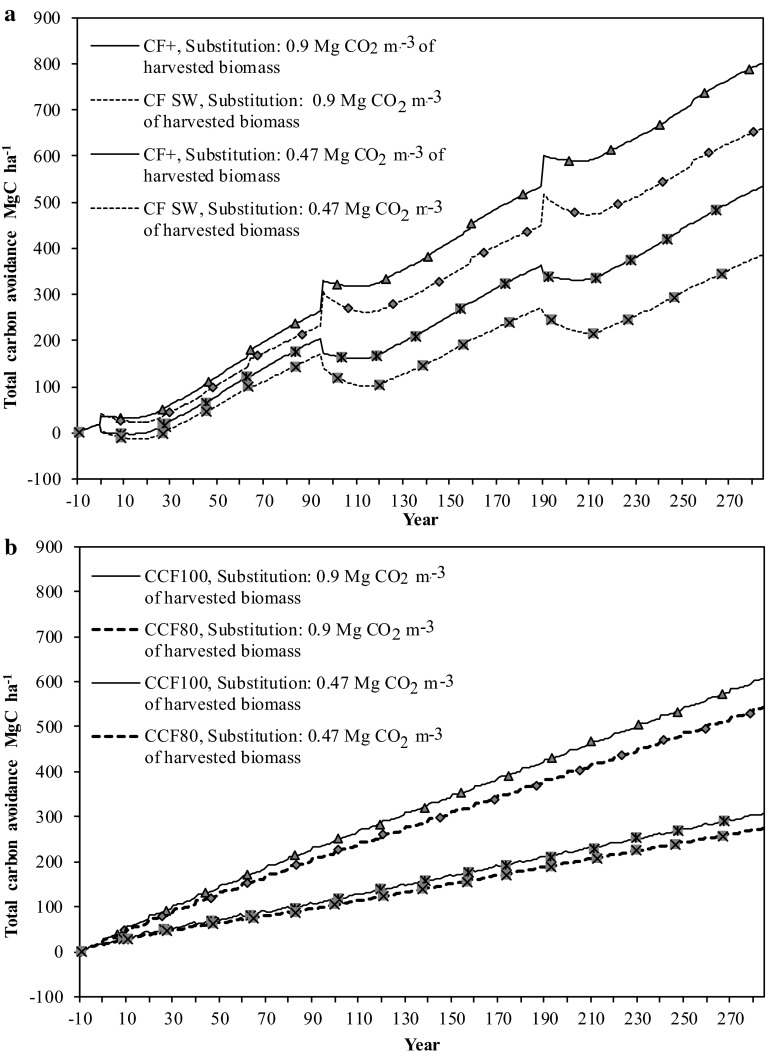
Cumulative total carbon balance for **a** *CF* **+** and *CF* and **b** *CCF100* and *CCF80* scenarios over 285 years with the implications of different substitution levels. *SW* stem-wood, *WT* whole-tree, and coal stands for coal reference fuel

When assuming a lower substitution effect (0.47 Mg CO_2_-eqv for each cubic meter of harvested biomass), the average annual net carbon balance dropped to 1.81, 1.29, 1.02, and 0.91 Mg C ha^−1^ year^−1^ for *CF*+, *CF*, *CCF100*, and *CCF80* scenarios corresponding to 50–60 % of the values obtained when assuming the higher substitution effect (Fig. 3a, b). Also with the lower substitution effect it remained the single most important component of the long-term climate benefit.

## Discussion

The results show that biomass growth is more important than the choice of silvicultural system per se. When comparing the two scenarios *CF* and *CCF100*, representing two principally different silvicultural systems, but with similar growth, extraction, and product use, only minor differences were found (Table 4). The importance of forest growth and long-term sustainable yields have been shown important for climate benefit in a number of other studies (Poudel et al. 2012; Lundmark et al. 2014; Kilpeläinen et al. 2015; Torssonen et al. 2015) supporting this conclusion. Also in this study, the effect of forest growth and yield was obvious especially when comparing the two CCF scenarios where the climate benefit was lower for the scenario with a lower average growth rate (Table 4).

We have compared the climate benefit of CF and CCF systems where the spatial boundary has been limited to the stand level. As a starting point for the analyses, we assumed the existence of a relatively heterogeneous spruce stand with two management alternatives for future land use. We restricted the period of analyses to 1 year before the third clear-cut in CF, after 285 years, and 1 year before the last selective cutting in CCF, after 290 years. By doing this, a large share of the growth from the third rotation period in CF is not harvested with the consequence that the average carbon stock change in the CF scenarios becomes higher than it should have been if the third clear-cut would have also been included. This explains why annual average change in standing forest carbon stock was higher for CF than for CCF in this analysis. On the other hand, the substitution effect became smaller than if the third clear-cut would have also been included. For the *CCF100* scenario, with comparable growth and extraction as the *CF* scenario, a larger share of the annual growth has been harvested giving lower average carbon stock change figures but corresponding greater impact of substitution (Table 4).

Analyzing the various parts of the carbon balance in the different scenarios reveals that the most important component was the substitution effect (Fig. 2). When assuming a high substitution level, the substitution effect accounted for more than 70 % of the total annual average carbon balance in all scenarios, stressing the importance of product use strategy and the availability of forests biomass for the total climate benefit of forestry. Also with the lower substitution level used in this study, the substitution effect was the most important component of the carbon balance. As a consequence, the largest climate benefit was achieved with *CF*+ because of whole-tree use (higher extraction level) and smallest with *CCF80* because of the lower production level (Figs. 2, 3). Assuming two different product use strategies, one representing a scenario where forest biomass is mainly used for energy and construction purposes (high substitution effect) and one which represents the current product use in Sweden, showed that the total future climate benefit may vary from 0.91 to 2.75 Mg C ha^−1^ year^−1^ (Fig. 3). If the CF scenario with a substitution effect of 0.47 Mg CO_2_-eqv for each cubic meter of harvested biomass is considered as the business as usual scenario, it can be concluded that the future climate benefit of forestry can increase or decrease relative to the present climate benefit of 1.29 Mg C ha^−1^ year^−1^ depending on the future growth and extraction rate of the managed forests and the use of extracted biomass. In order to make additional climate benefits compared with today, the most efficient strategy for the Nordic forests is to increase growth and yield and to maximize the substitution benefit. This can serve as a policy insight for coming discussions about future forest management and product utilization strategies in the context of climate change mitigation.

The simulation approach included a different set of models for CF and CCF. The Heureka system has been shown to give reliable results in traditionally managed Norway spruce stands (Fahlvik et al. 2014). Representation of CCF in the data behind the growth models in the Heureka is however limited. To better simulate the transition of trees between diameter classes in CCF it was decided to use growth models specifically developed for CCF (Chrimes and Lundqvist 2004). The models were based on six experimental plots within a small geographical area, which limited the scope of the simulations. To ensure that model dependent differences of the estimated growth level were not introduced, the production level in the CCF scenarios was adjusted according to scenarios (see “Materials and methods” section).

In the present study, it was assumed that a reverse J-shaped diameter distribution could be retained over a long period of time according to the model approach used. A sustainable J-shaped distribution is dependent on sufficient ingrowth to compensate for mortality and harvest. The ingrowth used in the simulations was comparable with levels found in field experiments in northern and central Sweden (Lundqvist 1993; Lundqvist et al. 2007). Lähde et al. (2010) studied a spruce dominated stand in Finland and found that the initial reversed J-shape diameter distribution remained 15 growing seasons after selection cutting. In a study by Ahlström and Lundqvist (2015) on uneven-aged spruce-dominated stands, it was concluded that it is possible to both maintain and restore a reverse J-shaped diameter distribution after harvest in heterogeneous spruce stands. Hence, these studies support the assumptions made about the sustained diameter distribution used in the CCF scenarios.

Comparative studies on mortality in CCF and CF are missing, and mortality in CCF was adjusted to equal the mortality proportion out of MAI in CF. Probability of mortality decreased with increasing DBH in the simulations which is in accordance to Pukkala et al. (2009). However, mortality in terms of proportion of stem number was lower than that found within experiments on uneven-aged spruce in central Sweden (Lundqvist 1993; Lundqvist et al. 2007).

The ambiguous results from studies of soil carbon in boreal forests, made us choose to not make different assumptions about older soil carbon development in the different scenarios. This can be questioned since the decomposition and mineralization of different carbon pools can be affected by disturbances like harvesting and also by environmental changes due to silvicultural measures such as thinning and clear-cutting. In this respect, the soil carbon pool may have been somewhat overestimated in the clear-cut scenarios, especially in the *CF*+ scenario where residues and stumps were also harvested (Egnell et al. 2015). Since soil carbon stocks are largely affected by forest growth (i.e., carbon input) the soil carbon pool may also have been overestimated in the *CCF80* scenario due to the assumed lower growth rates. We also used a constant decomposition rate for the estimate of litter carbon remaining in the forest for all scenarios (Table 3). Since litter production is influenced by forest growth and decomposition of litter by factors such as temperature and humidity this assumption might also have led to some differences between scenarios that have not been identified. The analyses of sensitivity to increased or decreased decomposition rates showed however that the effects were small in terms of annual average change in litter carbon stock and consequently did not influence the total carbon balance. These uncertainties regarding soil carbon together with the conflicting results from the various studies call for more studies on soil and litter carbon dynamics in relation to different silvicultural systems and within those different applied silvicultural programs. We recognize the uncertainties in our estimates of carbon stocks in the soil for the various scenarios, but we argue that differences that might not have been captured between the scenarios in this regard would not be decisive for the results.

## Conclusions

The choice of a silvicultural system per se was not important for the climate benefit. Instead, forest growth and yield together with the product use strategy determined the long-term climate benefit of forestry when analyzed at the stand level. In the long run, carbon stock changes in standing biomass, litter, and products were very small in managed forest systems as the ones used in this study when growth were assumed to be equal between the systems. As a consequence, the long-term average growth and yield will be more critical when discussing the silvicultural systems CF versus CCF, than other factors affecting the climate benefit of forestry.

## Electronic supplementary material

Supplementary material 1 (PDF 136 kb)

## References

[CR1] Ahlström M, Lundqvist L (2015). Stand development during 16–57 years in partially harvested sub-alpine uneven-aged Norway spruce stands reconstructed from increment cores. Forest Ecology and Management.

[CR2] Andreassen K, Øyen BH (2002). Economic consequences of three silvicultural methods in uneven-aged mature coastal spruce forests of central Norway. Forestry.

[CR3] Anon. 2014a. Swedish Forest Agency. Swedish Statistical Yearbook of Forestry. Available from www.skogsstyrelsen.se/statistics.

[CR4] Anon. 2014b. The Finnish statistical yearbook of forestry. Available from www.luke.fi/statistics.

[CR5] Assmann E, Assmann E (1970). The principles of forest yield study. Studies in the organic production, structure, increment and yield of forest stands.

[CR6] Bengtsson, G. 1978*. Beräkning av den naturliga avgången i avverkningsberäkningarna för 1973 års skogsutrednings slutbetänkande* [Calculation of natural loss in felling estimates for the 1973 forest investigation’s final report]. No. SOU 1978:7, Stockholm (in Swedish).

[CR7] Canadell JG, Raupach MR (2008). Managing forests for climate change mitigation. Science.

[CR8] Chrimes, D., and L. Lundqvist. 2004. Simulated volume increment of managed uneven-aged *Picea abies* stands in central Sweden. Paper 1 in *Stand development and regeneration dynamics of managed uneven*-*aged* Picea abies *forests in boreal Sweden*. Thesis, Acta Universitatis Agriculturae SUECIA, *Silvestria* 304, SLU.

[CR9] Clarke N, Gundersen P, Jönsson-Belyazid U, Kjønaas OJ, Persson T, Sigurdsson BH, Stupak I, Vesterdal L (2015). Influence of different tree-harvesting intensities on forest soil carbon stocks in boreal and northern temperate forest ecosystems. Forest Ecology and Management.

[CR10] Diochon A, Kellman L, Beltrami H (2009). Looking deeper: An investigation of soil carbon losses following harvesting from a managed northeastern red spruce (*Picea rubens* Sarg.) forest chronosequence. Forest Ecology and Management.

[CR11] Egnell G, Jurevics A, Peichl M (2015). Negative effects of stem and stump harvest and deep soil cultivation on the soil carbon and nitrogen pools are mitigated by enhanced tree growth. Forest Ecology and Management.

[CR12] Elfving, B. 1982. *Hugins ungskogstaxering* [Hugin’s Young Forest Inventory] *1976*–*1979*. In Faculty of Forestry, Swedish University of Agricultural Sciences, Umeå. Projekt Hugin, Report No. 27, 87 pp. (in Swedish).

[CR13] Elfving, B. 2006. Produktion vid byte från trakthyggen till blädning. In *Trakthyggesbruk och kontinuitetsskogsbruk med gran, en jämförande studie* [Even-aged stand system for Norway spruce vs continuous-cover forestry—A comparative study], ed. B. Karlsson. Skogsforsk, Redogörelse, ISSN 1103-4580 (in Swedish).

[CR14] Elfving, B. 2010. *Growth modelling in the Heureka system*. Swedish University of Agricultural Sciences, Faculty of Forestry. Retrieved October 1, 2015, from http://heurekaslu.org/wiki/Heureka_prognossystem_(Elfving_rapportutkast).pdf.

[CR15] Fahlvik, N., B. Elfving, and P. Wikström. 2014. Evaluation of growth models used in the Swedish Forest Planning System Heureka. *Silva Fennica* 48: article id 1013.

[CR16] Fridman J, Ståhl G (2001). A three-step approach for modelling tree mortality in Swedish forests. Scandinavian Journal of Forest Research.

[CR17] Gadow K, Gadow K, Nagel J, Saborowski J (2001). Orientation and control in CCF systems. International IUFRO conference on continuous cover forestry. Assessment, analysis, scenarios.

[CR18] Gustavsson L, Sathre R (2011). Energy and CO2 analysis of wood substitution in construction. Climatic Change.

[CR19] Gustavsson L, Haus S, Ortiz CA, Sathre R, Le Truong N (2015). Climate effects of bioenergy from forest residues in comparison to fossil energy. Applied Energy.

[CR20] Hägglund, B., and J.E. Lundmark. 1981. *Handledning i Bonitering med Skogshögskolans Boniteringssystem* [Tutoring in Site Quality Assessment with Forest School Assessment System]. Skogsstyrelsen, Jönköping (in Swedish).

[CR21] Holmgren, A. 1914. *Blädning och trakthuggning i Norrlandsskogar*. [Selection cutting and cutting treatments in northern forests]. Norrlands Skogsvårdsförbunds Tidskrift, 266–323 (in Swedish).

[CR22] Hyvönen R, Ågren GI (2001). Decomposer invasion rate, decomposer growth rate, and substrate chemical quality: How they influence soil organic matter turnover. Canadian Journal of Forest Research.

[CR23] Hyvönen R, Ågren GI, Linder S, Persson T, Cotrufo MF, Ekblad A, Freeman M, Grelle A (2007). The likely impact of elevated [CO_2_], nitrogen deposition, increased temperature and management on carbon sequestration in temperate and boreal forest ecosystems: A literature review. New Phytologist.

[CR24] Jandl R, Lindner M, Vesterdal L, Bauwens B, Baritz R, Hagedorn F, Johnson DW, Minkkinen K (2007). How strongly can forest management influence soil carbon sequestration?. Geoderma.

[CR25] Johnson DW, Curtis PS (2001). Effects of forest management on soil C and N storage: Meta analysis. Forest Ecology and Management.

[CR26] Jurgensen M, Tarpey R, Pickens J, Kolka R, Palik B (2012). Long-term effect of silvicultural thinnings on soil carbon and nitrogen pools. Soil Science Society of America Journal.

[CR27] Kilpeläinen A, Torssonen P, Strandman H, Kellomäki S, Asikainen A, Peltola H (2015). Net climate impacts of forest biomass production and utilization in managed boreal forests. GCB Bioenergy.

[CR28] Kreutzweiser DP, Hazlett PW, Gunn JM (2008). Logging impacts on the biogeochemistry of boreal forest soils and nutrient export to aquatic systems: A review. Environmental Reviews.

[CR29] Kuuluvainen T, Tahvonen O, Aakala T (2012). Even-aged and uneven-aged forest management in boreal Fennoscandia: A review. Ambio.

[CR30] Lähde E, Laiho O, Lin CJ (2010). Silvicultural alternatives in an uneven-sized forest dominated by *Picea abies*. Journal of Forest Research.

[CR31] Lindroth, A., P. Vestin, E. Sundqvist, M. Mölder, A. Bâth, M. Hellström, and P. Weslien. 2012. Clear-cutting is causing large emissions of greenhouse gases-are there other harvest options that can avoid these emissions? In *EGU general assembly conference abstracts*, vol. 14, p. 7578.

[CR32] Lundmark T, Bergh J, Hofer P, Lundström A, Nordin A, Poudel BC, Sathre R, Taverna R (2014). Potential roles of Swedish forestry in the context of climate change mitigation. Forests.

[CR33] Lundqvist, L. 1989. *Blädning i granskog*—*strukturförändringar, volymtillväxt, inväxning och föryngring på försöksytor skötta med stamvis blädning* [Selection cutting in spruce-structural changes, volume growth, ingrowth and regeneration in experimental plots managed by selective cutting system]. Sveriges Lantbruksuniversitet, Institutionen för skogsskötsel, Avhandling (in Swedish, English chapters).

[CR34] Lundqvist L (1993). Changes in the stand structure on permanent *Picea abies* plots managed with single-tree selection. Scandinavian Journal of Forest Research.

[CR35] Lundqvist L, Chrimes D, Elfving B, Mörling T, Valinger E (2007). Stand development after different thinnings in two uneven-aged *Picea abies* forests in Sweden. Forest Ecology and Management.

[CR36] Malmsheimer RW, Heffernan P, Brink S, Crandall D, Deneke F, Galik C, Gee E, Helms J (2008). Forest management solutions for mitigating climate change in the United States. Journal of Forestry.

[CR37] Marklund, L.G. 1988. *Biomassafunktioner för tall, gran och björk i Sverige* [Biomass functions for pine, spruce and birch in Sweden]. Sveriges Lantbruksuniversitet, Institutionen för Skogstaxering, Rapport 45. ISSN 0348-0496 (in Swedish).

[CR38] Melin Y, Petersson H, Nordfjell T (2009). Decomposition of stump and root systems of Norway spruce in Sweden—A modelling approach. Forest Ecology and Management.

[CR39] Möller, A. 1922. *Wie der Dauerwaldgedanke entstand*. In Der Dauerwaldgedanke [As the permanent forest idea arises], 5–18. Berlin: Springer (in German).

[CR40] Möller CM, Müller D, Nielsen J (1954). Graphic representation of dry matter production in European beech. Forstlige Forsoegsvaesen i Danmark.

[CR41] Nabuurs GJ, Masera O, Metz B, Bosch PR, Dave R, Meyer LA (2007). Chapter 9: Forestry. Climate Change 2007: Mitigation of Climate Change. Contribution of Working Group III to the Fourth Assessment Report of the Intergovernmental Panel on Climate Change.

[CR42] Näslund, B.-Å. 1986. *Simulation of damage and mortality in young stands and associated stand development effects (Rep. 18)*. Umeå: Department of Silviculture, Swedish University of Agricultural Sciences (in Swedish, English summary).

[CR43] Nilsson U, Fahlvik N, Johansson U, Lundström A, Rosvall O (2011). Simulation of the effect of intensive forest management on forest production in Sweden. Forests.

[CR44] Nyström, K., and U. Söderberg. 1987. *Tillväxtberäkningen för ungskog i HUGIN*-*systemet* [Growth calculations for young forests within the HUGIN-system]. En kontroll med data från återinventerade ungskogsytor. Institutionen för skogsskötsel. Sveriges Lantbruksuniversitet. Arbetsrapport 18 (in Swedish).

[CR45] Piirainen S, Finér L, Starr M (2015). Changes in forest floor and mineral soil carbon and nitrogen stocks in a boreal forest after clear-cutting and mechanical site preparation. European Journal of Soil Science.

[CR46] Poudel BC, Sathre R, Gustavsson L, Bergh J, Lundström A, Hyvönen R (2011). Effects of climate change on biomass production and substitution in north-central Sweden. Biomass and Bioenergy.

[CR47] Poudel BC, Sathre R, Bergh J, Gustavsson L, Lundström A, Hyvönen R (2012). Potential effects of intensive forestry on biomass production and total carbon balance in north-central Sweden. Environmental Science & Policy.

[CR48] Pukkala T (2014). Does biofuel harvesting and continuous cover management increase carbon sequestration?. Forest Policy and Economics.

[CR49] Pukkala T, Lähde E, Laiho O (2009). Growth and yield models for uneven-sized forest stands in Finland. Forest Ecology and Management.

[CR50] Pukkala T, Lähde E, Laiho O, Pukkala T, Gadow K (2012). Continuous cover forestry in Finland—Recent research results. Continuous cover forestry.

[CR51] Sathre R, O’Connor J (2010). Meta-analysis of greenhouse gas displacement factors of wood product substitution. Environmental Science & Policy.

[CR52] Shorohova E, Kapitsa E, Vanha-Majamaa I (2008). Decomposition of stumps in a chronosequence after clear-felling vs. clear-felling with prescribed burning in a southern boreal forest in Finland. Forest Ecology and Management.

[CR53] Sievänen R, Salminen O, Lehtonen A, Ojanen P, Liski J, Ruosteenoja K, Toumi M (2014). Carbon stock changes of forest land in Finland under different levels of wood use and climate change. Annals of Forest Science.

[CR54] Smyth CE, Stinson G, Neilson E, Lemprière TC, Hafer M, Rampley GJ, Kurz WA (2014). Quantifying the biophysical climate change mitigation potential of Canada’s forest sector. Biogeosciences.

[CR55] Tahvonen O (2009). Optimal choice between even-and uneven-aged forestry. Natural Resource Modeling.

[CR56] Thiffault E, Hannam KD, Paré D, Titus BD, Hazlett PW, Maynard DG, Brais S (2011). Effects of forest biomass harvesting on soil productivity in boreal and temperate forests—A review. Environmental Reviews.

[CR57] Torssonen P, Kilpeläinen A, Strandman H, Kellomäki S, Jylhä K, Asikainen A, Peltola H (2015). Effects of climate change and management on net climate impacts of production and utilization of energy biomass in Norway spruce with stable age-class distribution. GCB Bioenergy.

[CR58] Troup RS (1928). Silvicultural systems.

[CR59] Wallmo, U. 1897. *Rationell skogsafverkning: praktiska råd till såväl större som mindre enskilde skogsägare samt svar på en fråga för dagen* [Rational Forest Felling: practical advice for both larger and smaller individual forest owners and response to a question of the day], Stockholm 1897 (in Swedish).

[CR60] Wikström P, Edenius L, Elfving B, Eriksson LO, Lämås T, Sonesson J, Öhman K, Wallerman J (2011). The Heureka forestry decision support system: An overview. Mathematical and Computational Forestry & Natural-Resource Sciences (MCFNS).

[CR61] Yatskov M, Harmon ME, Krankina ON (2003). A chronosequence of wood decomposition in the boreal forests of Russia. Canadian Journal of Forest Research.

[CR62] Yrjölä T (2002). Forest management guidelines and practices in Finland, Sweden and Norway.

