# Supplementary material for: Comparison of carbon balances between continuous-cover and clear-cut forestry in Sweden
Source: Ambio. 2016 Jan 7;45(Suppl 2):203–13. doi: 10.1007/s13280-015-0756-3 (PMC4705068; doi:10.1007/s13280-015-0756-3)
Supplement: Supplementary file 1 — Supplementary material 1 (PDF 136 kb) [file 13280_2015_756_MOESM1_ESM.pdf]

Ambio

Electronic supplementary material

This supplementary material has not been peer reviewed

Title: **Comparison of carbon balances between continuous-cover and clear-cut forestry in Sweden**

Authors: Tomas Lundmark, Johan Bergh, Annika Nordin, Nils Fahlvik, Bishnu Poudel

Supplementary material 1

Forest growth and harvest modeling for Continuous-cover forestry

Clear-cut forestry (CF)

Stand development in CF was simulated with the Heureka system described by Elfving (2010), Fahlvik et al. (2014) and Wikström et al. (2011). Variables describing the site (latitude, altitude, site index, vegetation type), the stand (stand age, number of stems) and the individual trees (species, diameter and height) were input into the simulation There are two key growth stages in Heureka models: the stand establishment period (during which the average tree height within the stand is <7 m) and the development of established stands (average height >7 m). Stand development was predicted over a series of 5-year periods. During stand establishment, height growth was estimated using functions developed by Elfving (1982), and height-diameter relationships were defined based on Nyström and Söderberg (1987). Estimates of mortality and damage in the young forest were based on functions developed by Näslund (1986). In the established stand (average height >7 m), a different set of growth functions was applied. Basal area growth was calculated by combining distance-independent functions for single trees and a stand-level function (Elfving 2010). The latter function was used to calibrate the growth level while the individual-tree functions were used to distribute growth on individual trees. The annual mortality in percent of standing volume was estimated with functions developed by Bengtsson (1978), and functions by Fridman and Ståhl (2001) were used to distribute the mortality along the dimension distribution. Output tree data from Heureka were used to calculate biomass of whole trees as well as the fractions of trees (stem, branches, needles and roots) according to Marklund (1988).

Two scenarios with CF were assumed; one where only stem wood was harvested (*CF*) and the other with a higher degree of extraction where 80% of the residues and stumps after clear-cut was also harvested (*CF+*). The CF scenario could also be seen as the business as usual scenario.

Continuous-cover forestry (CCF)

Stand development in CCF mainly relied on models developed by Chrimes and Lundqvist (2004). The models were based on six experimental plots within Siljansfors Experimental Forest in central Sweden (60° 53´ N, 14° 25´ E, 300 m a.s.l). Single-tree selective harvests had been applied within the six stands and the initial diameter distributions resembled a reverse-J shaped curve on most of the plots. Only trees with a diameter at breast height (1.3 m; DBH) greater than 8.5 cm were used for the development of the models. The diameter distribution was divided into 2 cm classes and the stand development was simulated in periods of 5 years. Chrimes and Lundqvist (2004) did not include mortality as a part of the growth modeling. In the present study the mortality was simulated with functions by Fridman and Ståhl (2001) and was adjusted to equal the mortality generated in the simulations of CF. Selection harvest of all trees with DBH class 35.5 and greater was carried out every 10 years. Simulations were set to correspond to two predefined scenarios of CCF; CCF with mean annual volume increment (MAI) corresponding to 80% (*CCF80*) and 100% (*CCF100*) of *CF* at equilibrium, respectively. The proportion of mortality out of total volume production in CCF was also set equal to CF. Equilibrium in CCF was met when ingrowth compensated for harvest and mortality and identical 10 year cycles were repeated over time. The simulations followed seven steps:

1. Mortality. Function by Fridman and Ståhl (2001) for Norway spruce were used to distribute mortality along the diameter distribution. Fridman and Ståhl (2001) developed functions to calculate the probability of single tree to die during a 5 years period:

$$P_{mort} = \frac{e^{a+b'X}}{1+e^{a+b'X}}$$

where P_{mort} is the probability for a tree to die (0-1), a and b are parameters and X the independent variables including expressions for DBH of the subject tree, mean DBH, total basal area, basal area of larger trees, time since thinning and species distribution. P_{mort} was calculated for each DBH class and mortality was distributed over the DBH classes according to:

$$N_{mort_i} = N_i \times P_{mort_i} \times A_{mort}$$

Where i is an index for DBH class, N_{mort} is the number of dead trees, P_{mort} is the probability of mortality and A_{mort} is a multiplier with the same value applied on all DBH classes.

2. Growth. Basal area growth of single trees within each DBH class was estimated with function by Chrimes and Lundqvist (2004) according to:

$$\ln(\mathrm{iBa}) = a + b'X$$

Where iBa is 5 years basal area growth, a is the intercept, b is parameters and X is independent variables including expressions of class mean DBH, total basal area on the plot and basal area of overtopping trees. Different growth levels in CCF80 and CCF100 where achieved by adding a multiplier to the estimated basal area growth function; $iBa_{adj}=iBa*A_{growth}$. The same value of A_{growth} was used for all DBH classes.

3. Upgrowth and ingrowth. DBH of living trees within each class was updated based on the basal area growth (iBa_{adj}) and upgrowth into greater diameter classes was calculated. Ingrowth into the smallest diameter class was input to the simulator and was adjusted to achieve an equilibrium state where ingrowth compensated for mortality and harvest.

4. Tree height and form height. Mean height for each DBH class was estimated using the height-diameter relationship by Chrimes and Lundqvist (2004). Form height for each DBH class was calculated according to Chrimes and Lundqvist (2004) as hf=a+b´X; where hf is the form height, a is the intercept and b is parameters and X is independent variables including expressions of class mean DBH and total basal area.

5. Small trees. Chrimes and Lundqvist (2004) did not include trees with DBH <8.5 cm. A polynomial function was fitted to the equilibrium DBH >8.5 cm diameter distribution in order to extrapolate the number of stems to DBH classes 1.5 to 7.5 cm. Mean height within DBH class 1.5 to 7.5 was estimated by extrapolating the height-diameter relationship by Chrimes and Lundqvist (2004).

6. Volume and biomass. Volume within each class was calculated by multiplying form height with basal area corresponding to class mean DBH and the number of stems. Class mean DBH and height were input to Marklund (1988) to estimate above and below ground biomass within each diameter class. Volume and biomass was calculated separately for living and dead trees.

7. Harvest: All living trees within DBH class 35.5 and greater were harvested and removed from the DBH distribution every second 5 years period.

Ingrowth, A_{mort} and A_{growth} were adjusted to reach equilibrium stand development corresponding to the predefined volume production and mortality rate in CCF80 and CCF100.

Simulations

Site conditions

The geographical location and site factors were selected in order to correspond to the data used for parameterization of the CCF growth model (Chrimes and Lundqvist 2004). The mean annual temperature of the area was +5 °C and the mean annual precipitation was 900 mm. The soil moisture was mesic and the field layer was dominated by bilberry (*Vaccinium myrtillus*). The site index based on site factors was 24 m, defined as the dominant height at a total age of 100 years for Norway spruce (Hägglund and Lundmark 1981).

Initial stand

The initial stand, 10 years before the establishment of the different scenarios and the start of the simulations, had 515 stems ha^{-1} with DBH>8.5 cm, a standing volume of 130 m^3 ha^{-1} and an average annual growth of 5.6 m^3 ha^{-1} $year^{-1}$. After 10 years, at the start of the simulations, the number of stems with DBH>8.5 cm was 556 ha^{-1}, the basal area was 20 m^2

ha^{-1} and the standing volume was 182 m^3 ha^{-1}. The simulation started in the year 0 and we assumed four different futures for the stand, i.e. the different scenarios used.

CF

After clear-cut of the original stand a new Norway spruce stand was established. Due to difficulties to simulate the plant development stage, empirical field data from a young even-aged stand of Norway spruce was selected as input to Heureka. The stand was 13 years old with an average tree height of 2.5 m and situated on a similar site as the plots used by Chrimes and Lundqvist (2004). Based on empirical field data a stand with 2000 stem ha^{-1} was reconstructed which corresponds to a typical planting spacing in central Sweden. We assumed natural regeneration to be negligible and that there was no need for pre-commercial thinning. Two thinnings from below with a thinning grade of 26 and 30%, respectively, of the basal area were carried out at an age of 45 and 65 years. The basal areas before first and second thinning were 25.5 and 34 m^2 ha^{-1} respectively. The total volume removed at thinning was 134 m^3 ha^{-1}. Clear cutting was carried out at a total stand age of 95 years. The number of stems at final felling was 650 ha^{-1} and the standing volume was 497 m^3 ha^{-1}. The MAI during the rotation was 7.0 m^3 ha^{-1} year^{-1} and the mortality was 5.6% of the total production. Also the development from planting until 2.5 m average tree height was considered in the overall calculations of CF.

CCF

Both CCF80 and CCF100 aimed at a mean standing volume of 150 m^3 ha^{-1}. After repeated simulations, a balance between harvest, mortality and ingrowth was found when annual ingrowth into the 8.5 cm diameter class was set to 5.5 and 6.5 trees ha^{-1} for CCF80 and CCF100, respectively resulting in slightly different diameter distributions before harvest (Fig. 1). Mean annual increment (MAI) was 5.6 and 7.0 m^3 ha^{-1} $year^{-1}$ and the harvested volume each 10-year period was 53 and 67 m^3 ha^{-1} for CCF80 and CCF100, respectively. The basal area before each selection cut for CCF80 and CCF100 scenarios were 20.0 and 20.3 m^2 ha^{-1}. The selection cutting reduced the stand basal area by 23% and 28% in CCF80 and CCF100, respectively. Volume of trees with DBH<8.5 was 3–4% of the total standing volume. Mortality was 5.6% of total production in both scenarios. Average mortality in terms of stem number was 1.3 and 1.7% during a 5 years period for DBH>8.5 cm in CCF80 and CCF100 (Fig. 2).

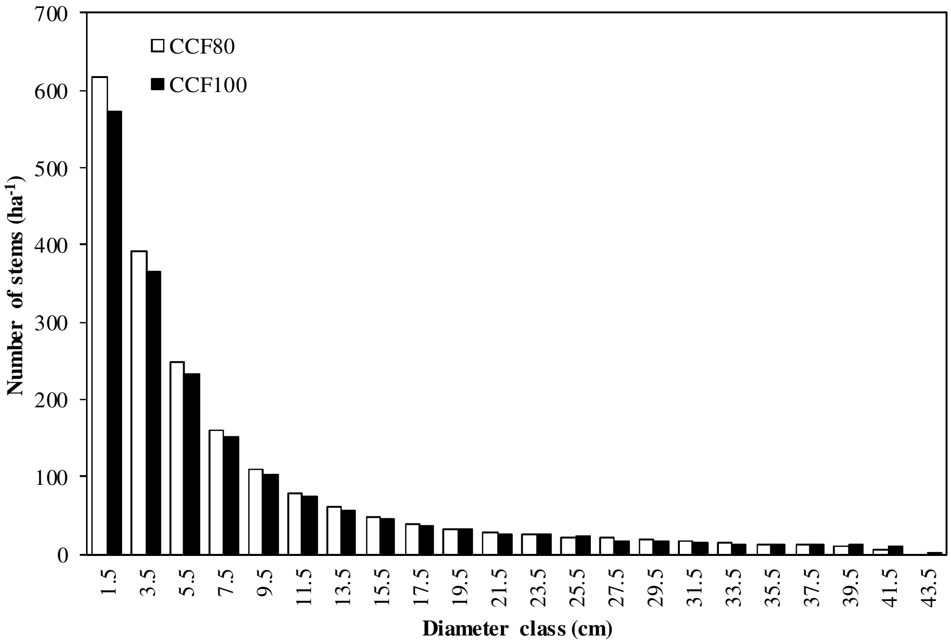

Fig. 1 Simulated diameter distributions before harvest in continuous cover forestry. Two scenarios included continuous-cover forestry with mean annual volume increment corresponding to 80% (*CCF80*) and 100% (*CCF100*) of a simulated clear-cut forestry (*CF*) on the same site

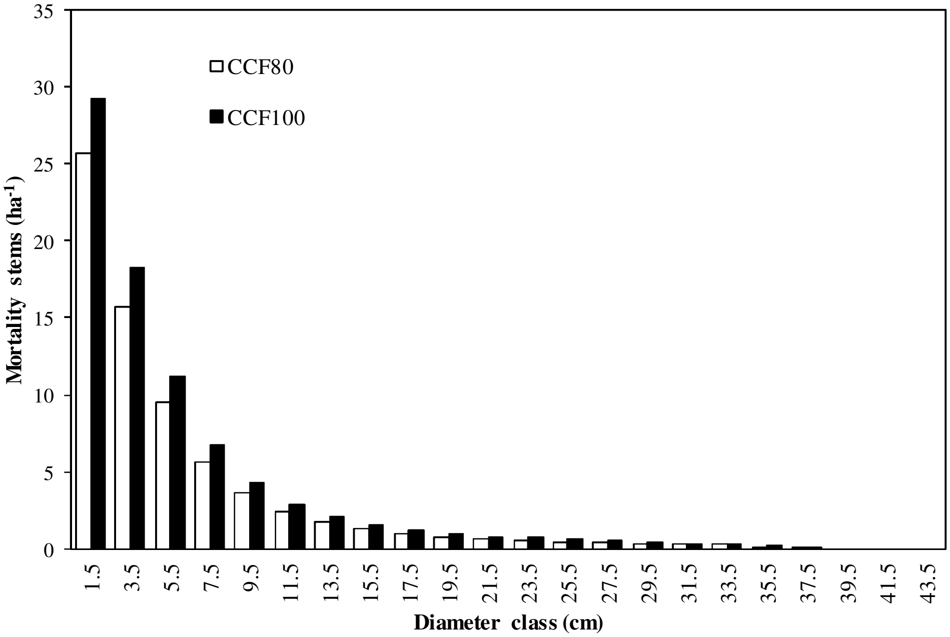

Fig. 2 Simulated mortality expressed as the number of dead stems within diameter classes during a harvesting cycle of 10 years. Two scenarios included continuous-cover forestry with mean annual volume increment corresponding to 80% (*CCF80*) and 100% (*CCF100*) of a simulated clear-cut forestry (*CF*) on the same site
